# Development of Proprioceptive Acuity in Typically Developing Children: Normative Data on Forearm Position Sense

**DOI:** 10.3389/fnhum.2016.00436

**Published:** 2016-08-29

**Authors:** Jessica M. Holst-Wolf, I-Ling Yeh, Jürgen Konczak

**Affiliations:** Human Sensorimotor Control Laboratory, School of Kinesiology, University of MinnesotaMinneapolis, MN, USA

**Keywords:** sensorimotor, proprioception, position sense, human, development

## Abstract

This study mapped the development of proprioception in healthy, typically developing children by objectively measuring forearm position sense acuity. We assessed position sense acuity in a cross-sectional sample of 308 children (5–17 years old; M/F = 127/181) and a reference group of 26 healthy adults (18–25 years old; M/F = 12/14) using a body-scalable bimanual manipulandum that allowed forearm flexion/extension in the horizontal plane. The non-dominant forearm was passively displaced to one of three target positions. Then participants actively matched the target limb position with their dominant forearm. Each of three positions was matched five times. Position error (PE), calculated as the mean difference between the angular positions of the matching and reference arms, measured position sense bias or systematic error. The respective standard deviation of the differences between the match and reference arm angular positions (SDP_diff_) indicated position sense precision or random error. The main results are as follows: First, systematic error, measured by PE, did not change significantly from early childhood to late adolescence (Median PE at 90° target: −2.85° in early childhood; −2.28° in adolescence; and 1.30° in adults). Second, response variability as measured by SDP_diff_ significantly decreased with age (Median SDP_diff_ at 90° target: 9.66° in early childhood; 5.30° in late adolescence; and 3.97° in adults). The data of this large cross-sectional sample of children document that proprioceptive development in typically developing children is characterized as an age-related improvement in precision, not as a development or change in bias. In other words, it is the reliability of the perceptual response that improves between early childhood and adulthood. This study provides normative data against which position sense acuity in pediatric patient populations can be compared. The underlying neurophysiological processes that could explain the observed proprioceptive development include changes in the tuning of muscle spindles at the spinal level, the maturation of supraspinal somatosensory pathways and the development of interhemispheric callosal connections responsible for the transfer of somatosensory information.

## Introduction

Proprioception refers to the sense of relative position and movement of the limbs and body (Konczak et al., 2009). Proprioceptive information is provided through mechanoreceptors embedded in the joints, muscles, tendons and skin. It has long been recognized that intact proprioception is essential for the control of muscle tone and voluntary movement. It is further known that pediatric conditions such as cerebral palsy, autism or developmental coordination disorder are associated with somatosensory or proprioceptive deficits that negatively affect movement control (Coleman et al., 2001; Kaufman and Schilling, 2007; Goble et al., 2009; Wang et al., 2009; Zwicker et al., 2013; Li et al., 2015).

In contrast to other senses such as vision or audition, the development of proprioception in typically developing children has never been fully mapped. Previous research has established that kinesthetic sensitivity and acuity improves in middle childhood and continues to approach adult levels during late adolescence (Laszlo and Bairstow, 1980; Bairstow and Laszlo, 1981; Elliott et al., 1988; Visser and Geuze, 2000; Goble et al., 2005; Pickett and Konczak, 2009). However, no consistent assessment protocol has been established. The available empirical results were either based on small samples, had methodological shortcomings, or were restricted to limited age groups that did not provide a comprehensive understanding of proprioceptive development across childhood and adolescence. Moreover, most reported results are not comparable between studies because of differences in protocol and test equipment.

One reason for the current lack of reliable normative data against which the proprioceptive status of pediatric populations can be compared, lies in the fact that proprioceptive function is not easily examined. Clinical rating scales are coarse and lack sufficient sensitivity, which allow only for detection of severe deficits in proprioception. Objective testing of proprioceptive function may require specialized equipment that is expensive and typically not available in clinical settings. In addition, testing takes too long or demands high levels of attention, which becomes problematic especially for younger children. Moreover, available kinesthetic acuity tests have failed to consistently differentiate pediatric populations from healthy children (Lord and Hulme, 1987; Hoare and Larkin, 1991; Piek and Coleman-Carman, 1995). Early work to characterize the development of proprioception by Laszlo and Bairstow (1980) produced the Kinesthetic Sensitivity Test. However, in this test, approximately 25% of the 5- and 6-year old participants never responded correctly for more than half of the time (Bairstow and Laszlo, 1981). This is equivalent to random guessing and illustrates the difficulty in designing a reliable and valid test of proprioceptive function useful for clinical assessment. Other available tests such as the Movement Assessment Battery for Children 2nd edition (Kirby and Sugden, 2007; Li et al., 2015) focus primarily on motor but not on proprioceptive deficits.

When designing a test of proprioceptive assessment for children, one needs to consider the following: first, which proprioceptive modality shall be examined: position or motion sense? Position sense testing is technically simpler, because it does not require an apparatus to control the velocity of limb motion. Second, will sensitivity or acuity be tested? (We here refer to *sensitivity* as the ability to detect a proprioceptive stimulus and *acuity* as the ability to discriminate between two detectable stimuli). Third, is the test duration short and simple enough to assure that even young children can complete it without being mentally and cognitively challenged? Fourth, does the test equipment conform to the large differences in anthropometrics that are observed during childhood?

Here, we attempted to address the above challenges. Based on our previous work (Elangovan et al., 2014) and the work of others (Goble et al., 2005; Goble, 2010), we opted to employ a contralateral arm matching task to measure position sense acuity in children. We designed a bimanual manipulandum that can be body-scaled and is able to conform to anthropometrics of children, adolescents, and adults, while providing highly precise objective measures of forearm position (and velocity). We selected a bimanual, contralateral matching task, because it removes any confounds related to memorizing a position or having to generate an internal representation of the limb position (Goble et al., 2005; Goble, 2010; Elangovan et al., 2014). The method generates objective measures of acuity in a relatively short time span and is thus ideal for assessing proprioceptive function in children.

**Figure 1:**
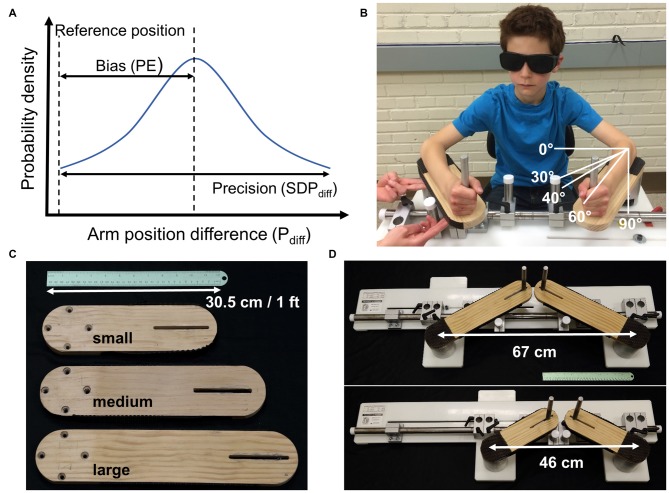
**(A)** Bias and precision are the two components of acuity. Shown is a hypothetical population or individual probability density function for limb position matching responses the difference between the match and reference arm angular positions (degrees; P_diff_). The reference position is the true position. Accuracy requires minimal or no bias (low position error (PE)) and high precision (low SDP_diff_). **(B)** Experimental setup with a participant seated in front of the bimanual manipulandum. Here the experimenter passively moved the non-dominant, right arm and the participant actively matched the position with the dominant left arm. The white lines depict the reference, or start position of 30° for the left arm as well as the three target positions. **(C)** Interchangeable lever arms accommodated a wide range of forearm lengths from children to adults fitting forearms lengths between 15 and 35 cm. **(D)** The left lever arm was adjustable to accommodate differences in shoulder width.

This study mapped the development of proprioceptive acuity in a large, cross-sectional sample of typically developing children with the aim to obtain a normative data set against which the proprioceptive status of pediatric populations can be compared. When envisioning the developmental time course of proprioceptive acuity, one needs to consider that acuity has two aspects: bias and precision (Figure 1A). Here bias indicates how close a sensed limb position corresponds to the true physical position of the forearm (I.S.O., 1994). For a true response, there is no systematic error or bias. Precision represents the random error or the agreement between independent repeated responses (I.S.O., 1994). This implies that during development either an initially high systematic error is reduced, or precision increases with age, or proprioceptive development is characterized by a combination of both.

## Materials and Methods

### Participants

Twenty-six healthy adults (M age: 20 years 11 months (SD: 1 year 8 months); 12 ♂, 14 ♀) and 308 children participated (age range: 5 years 4 months to 17 years 10 months; 127 ♂, 181 ♀). Recruitment and data collection occurred at a local primary school, on the University of Minnesota campus, and at the Minnesota State Fair. Appropriate parental consent and child assent was obtained before data collection. The study was approved by the University of Minnesota Internal Review Board. All subjects completed a modified Edinburgh handedness inventory to determine the dominant upper limb. Exclusion criteria for participation were the documented presence of central or peripheral nervous system disease, current injury to the upper limbs and/or implanted medical devices in the arms which may impair sensorimotor function.

### Apparatus

A bimanual manipulandum with one degree of freedom in the horizontal plane was used to perform the bilateral arm position matching task performed in this study (Figure 1). Two US Digital H6 optical encoders (2500 quadrature count/revolution; spatial resolution: 0.14°), housed at the rotating point of the manipulandum lever arms, recorded the angular position of each arm at a sampling frequency of 43 Hz.

### Procedure

Participants were seated, and they placed each arm on the bimanual levers. Chair height, lever arm length and handle placement were adjusted to the anthropometrics of each participant such that the approximate joint axis of the elbows aligned directly with encoder shaft axis. Distance between the two levers was adjusted such that the participants’ elbows were a comfortable distance apart (45°–85° of shoulder abduction). At the start of each trial and between target positions participants rested each arm at a start position set at 30° from the frontal plane (see Figure 1B). Participants wore vision occluding glasses during all trials and could not see their arms. The non-dominant arm was passively moved in the horizontal plane away from the body at a consistent speed of 20°–30°/s to one of three target positions, 40°, 60° or 90° from the frontal plane of the participant (see Figure 1B). We tested three different positions because displacing a limb to a higher amplitude is associated with a greater error (Goble et al., 2005) and we here sought to account for possible developmental differences of position sensing across the forearm workspace. Once the target position was reached, the participant moved the dominant arm to match the position of the other arm. Participants had as much time as desired to match the position. Participants verbally indicated when the matching position was achieved and held this position. Positions of each arm were then recorded for 1.6 s (collecting 70 samples of current limb position). Then the researcher moved the non-dominant hand back to the start position and gave a verbal cue for the participant to actively move the dominant arm back to the start position. Target positions were presented in pseudo random order such that each position was repeated 5 times for a total of 15 trials. One or two practice trials preceded data collection to familiarize the participants to the task and ensure that the instructions of the task were understood. If the researcher or child visibly moved during the 1.6 s recording, for example, if an arm was brought back to the start position early, the trial was repeated. Total testing time was approximately 10 min.

### Measurements

For each trial the physical position of each arm was determined by computing the average position over the recorded 70 samples (i.e., while holding the position). Subsequently, the average position of the matching arm was subtracted from the average position of the reference arm (P_diff_). Finally, for each participant, the position error (PE) was computed as the mean of P_diff_ across the five trials for each target position (40°, 60° and 90°). Similarly, the corresponding standard deviation was computed as the standard deviation (SDP_diff_) of P_diff_ across the five trials for each participant and target position. PE indicates a measure of bias or systematic error, while SDP_diff_ indicates the response precision across trials. Each participant’s chronological age at the date of data collection was calculated in years and months. Months were converted to base 10 (i.e., a year was subdivided into 10 sections).

### Results

**Figure 2:**
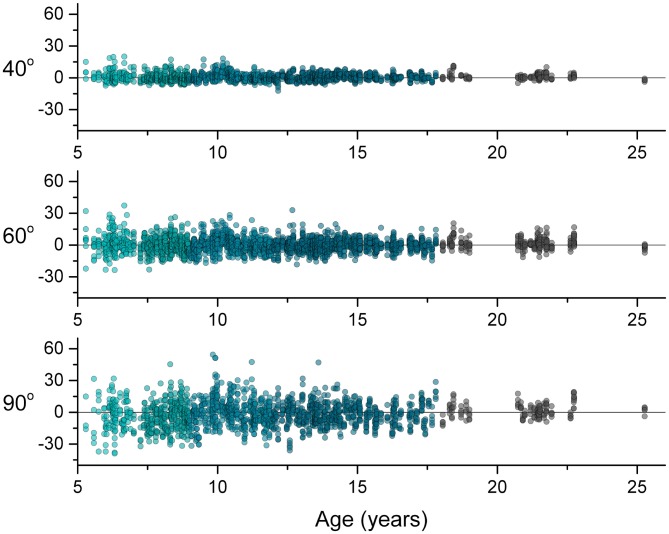
**Differences between arm positions (P_diff_) for each trial across age for each target positions.** A perfect forearm position match would have a P_diff_ value of zero, a positive value indicates overshooting and a negative value indicates undershooting. Note that the data distribute about zero across age for each target.

To characterize the breadth of response behavior across the development, Figure 2 shows the individual responses (P_diff_) of all 334 participants for each target position. Across the cross-sectional age sample, P_diff_ was distributed about zero degrees for each target position, meaning that there was no systematic age-related trend to undershoot (more flexed than the reference position) or overshoot (more extended than the reference position).

We further characterized the development of position sense acuity by investigating both bias and precision. To determine systematic changes in bias during development, a repeated measures analysis of variance (ANOVA) of PE was performed (chronological age × target position × gender × handedness). No significant main effects of PE for age, gender and handedness were found (*p*’s > 0.05). A significant interaction between age and target position was found (*p* = 0.048). However, linear regression procedures on PE by chronological age for the 40° and 60° target position did not reach significance (*p*’s > 0.05). The regression fit for the 90° target position reached significance (*p* = 0.04). However, the adjusted coefficient of determination for this linear model was low indicating that only age explained 1% of the variability in PE for the 90° target position (*R*^2^_adjusted_ = 0.01).

**Figure 3:**
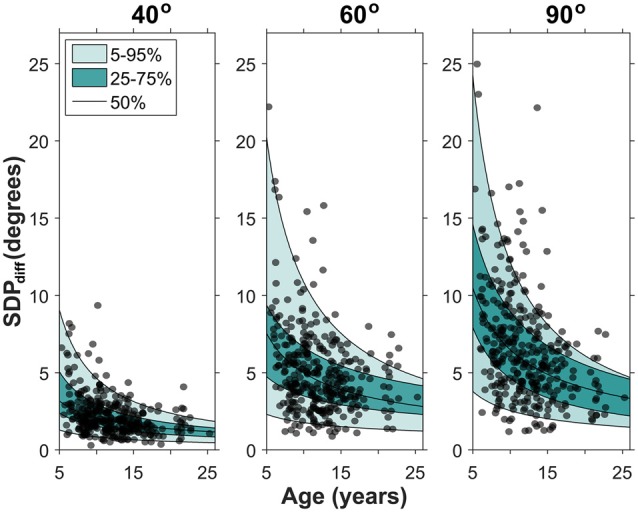
**Developmental changes in precision for each target position.** Each data point represents a participant’s SDP_diff_. The quantile regression estimates for the 5th, 25th, 50th, 75th and 95th percentile are shown. Note that SDP_diff_ decreased with age, indicating an age-related improvement in precision. The median fit for each target position is: SDP_diff,_40__ = 8.43 *age^−0.61^, SDP_diff,_60__ = 19.83 *age^−0.60^, SDP_diff,_90__ = 32.18 *age^−0.70^.

To determine developmental change in position sense precision, the corresponding SDP_diff_ data were analyzed. The distribution of the SDP_diff_ data was significantly different from normal which was corrected with a log_10_ transformation. A full model repeated measures ANOVA of the log transformed SDP_diff_ data (chronological age × target position × gender × handedness) was then performed. No significant main effects and interactions of gender and handedness were found (*p*’s > 0.05). The reduced model (chronological age × target position) revealed significant main effects of both amplitude (*p* < 0.001) and chronological age (*p* < 0.001), but no significant interaction. That is, the precision component of acuity changed with age and target position. Non-linear quantile regression procedures were conducted to estimate the SDP_diff_ percentiles across age for each target position. Figure 3 maps this age-related change in SDP_diff_ separately, for each target amplitude, illustrating that for each target SDP_diff_ decreased with increasing age.

**Figure 4:**
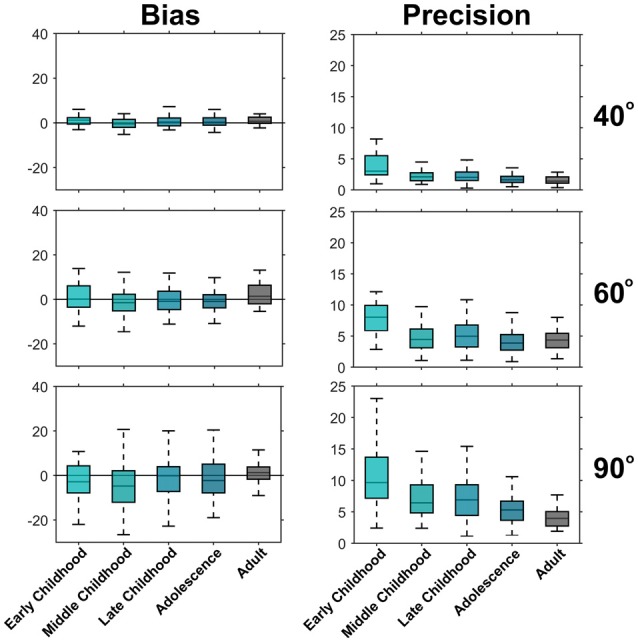
**Development of bias and precision across developmental periods.** Shown are boxplots for PE (left) and SDP_diff_ (right). The box lines indicate the 25th, 50th and 75th percentiles and the whiskers extend to the 5th and 95th percentile, outliers have been omitted for clarity. There is no trend of the median PE across development indicating no change in bias across age. However, the median SDP_diff_ for each age period decreases with increasing age indicating an improvement in precision during development. Early childhood (5–6 years old, *n* = 25), Middle childhood (7–8 years old, *n* = 62), Late Childhood (9–11 years old, *n* = 97), Adolescence (12–17 years old, *n* = 124), Adults (18–25 years old, *n* = 26).

Grouping participants into developmentally appropriate classes (Payne and Isaacs, 2008) underlines the assessment that there are minimal age-related differences in PE (see Figure 4). For example, median PE for the 90° target ranged between −4.8° and 1.3° with no age-related, systematic change in PE discernible. To appreciate the developmental trend toward increased precision, consider that at 5–6 years of age (early childhood) the 5% to 95% range of SDP_diff_ for the 90° target was 2.4°–23.0°, compared to 1.2°–10.6° in adolescence and dropped to 1.9°–7.7° in adulthood.

To investigate, if the age-related decrease in SDP_diff_ is a result of an age-related decrease in PE, we performed linear regression procedures for SDP_diff_ with PE as the predictor for each of the three target positions. All regression procedures yielded significant correlations (*R*^2^_adjusted_: 0.12 for 40°, 0.10 for 60°, and 0.05 for 90°; *p*’s < 0.05). However, no more than 12% of the age-related change in SDP_diff_ was explained by changes in PE, indicating that PE was not a strong predictor of SDP_diff_.

## Discussion

This is the first study to map proprioceptive development from early childhood to young adulthood in a large cross-sectional sample with an objective, quantitative measure. We employed a simple, child-appropriate method that conforms to a child’s anthropometrics and provides objective measures of forearm position sense acuity. Specifically, we systematically examined how bias and precision components of proprioceptive acuity changed during development. The main findings are that proprioceptive development between the ages of 5 and 18 years is not characterized as a development of decreasing bias, or inversely an increase in the accuracy of sensing. Instead, it is understood as a development where the precision improves with age. That is, the sensory response variability decreases with age. In summary, position sense does not become more accurate, but it does become more reliable during development.

### Neurophysiological Factors Underlying Proprioceptive Development

What are the possible neural correlates of proprioceptive development in our sample of children? First, the observed age-related change in precision is not likely due to maturation of peripheral proprioceptors. We examined children as young as 5 years of age, but muscle spindles, which contribute significantly to position sense, are known to be mature in children as young as 3 years of age (Österlund et al., 2011). Moreover, spinal level somatosensory circuitry is functional at a young age. This is demonstrated by findings that threshold amplitudes for eliciting stretch and Hoffman reflex responses reach adult levels by 6–7 years of age (O’Sullivan et al., 1991; Grosset et al., 2007).

Second, changes in muscle spindle sensitivity across age may in part explain the age related differences in position sense response variability. Research demonstrating that developmental changes in stretch response amplitude resulting from muscle spindle tuning by altered γ motoneuron activation (Grosset et al., 2007) underline this view. Given that muscle spindles and the relevant spinal circuitry are largely functional by middle childhood, developmental changes in spindle sensitivity would likely be influenced by changes in the descending signals projecting from supraspinal centers that target γ motorneurons which, in turn, innervate the intrafusal fibers of the spindles.

Third, developmental changes in proprioceptive precision could be influenced by the development of supraspinal structures involved in the inter-hemispheric transfer of somatosensory information such as the corpus callosum. This is plausible, because our limb position matching task required the transfer of position information of the reference limb to the opposite hemisphere to correctly position the matching arm (Goble et al., 2005; Goble, 2010). Thus, the development of callosal projections that enable the communication between brain hemispheres could also have influenced the perceptual outcome measure. Axon growth and axon cytoskeletal changes of the corpus callosum are known to occur throughout childhood progressing into adulthood (Keshavan et al., 2002; Lebel et al., 2010).

Finally, maturation of cortical structures responsible for somatosensory processing occurs at least until middle to late childhood. In typically developing children, the morphology of somatosensory evoked potentials (SEP) by median nerve stimulation is already mature by 3 years of age (Laget et al., 1976), but latencies are not mature until around 6–8 years of age (Boor et al., 1998; Boor and Goebel, 2000). This likely reflects developmental changes in the thalamo-cortical and lemniscal segments of somatosensory pathways. Contralateral somatosensory evoked fields (SEF) produced by index finger tactile stimulation become adult-like around the age of 2 years, while their morphology and latency continues to change through at least until 6 years of age (Pihko et al., 2009). Moreover, cortical changes such as maturation of the neurotransmitter system and axon and dendrite growth occur through adolescence (Nevalainen et al., 2014). Recent brain imaging examined how proprioceptive cortical networks develop in adolescence (Cignetti et al., 2016). Employing tendon vibration to stimulate muscle spindles which then induce illusory movement, the study demonstrated that the proprioceptive brain network is already firmly established prior to early adolescence but undergoes continued refinement as evidenced by a shift from diffuse to focal functional connectivity that was especially pronounced in fronto-striatal connections.

In summary, an array of neural changes influence signal generation at the receptor level and the subsequent processing of these signals in supraspinal networks. Given that muscle spindles are unique sensors, because their response sensitivity to muscle tension can be altered by descending input to γ motorneurons, the fine tuning of these γ motorneurons pools is plausibly a major determinant of proprioceptive development.

### Alternative Explanations and Limitations

This study employed a method in which participants had to match a given joint position by actively moving the forearm. This implies that an efference copy of the motor commands underlying such active movements was available to the nervous system to predict movement outcome. Thus, the sensing of joint position could have been influenced by at least three neural processes: the generation of internal predicted sensory feedback the processing of afferent sensory feedback and the integration of these two types of feedback to obtain a stable percept of limb position (Von Holst, 1954; Blakemore et al., 2001; Konczak et al., 2012). One therefore needs to recognize that this method does not provide a “pure” sensory measure of proprioceptive acuity (i.e., the sole processing of proprioceptive afferents). However, it is unlikely that the active movement to match a target position caused an unsystematic bias, across ages, that confounds the results. From a clinical perspective it may even be argued that the active movement testing represents a more functional scenario.

Finally, one needs to consider if developmental differences in motor control could account for the differences in the proprioceptive precision. While one cannot fully exclude this possibility, we had attempted to address this potential confound between motor ability and proprioceptive acuity by: (a) measuring forearm position at rest; and (b) by allowing participants as much time as needed to achieve their desired limb position. Thus, possible differences in movement kinematics (speed, smoothness) had little to no influence on our perceptual outcome measure.

In summary, the active joint position matching method provides a functional measure of a child’s proprioceptive status, but its results do not represent a measure of “pure” sensory proprioceptive acuity. It constitutes a trade-off between simplicity of testing and a higher measurement accuracy attainable through the use of specialized equipment such as a passive motion apparatus.

## Conclusion

Here we presented a simple, time- and cost-effective method to objectively measure proprioceptive acuity in children using a bimanual manipulandum that can be body-scaled to the anthropometric dimensions of the child. Assessing limb position sense acuity of the forearm in a large cross-sectional sample of children between the ages of 5 and 18 years revealed that the development of proprioceptive acuity in typically developing children is not characterized by a change in systematic bias but rather is associated with an increase in precision with increasing age. Several neurodevelopmental processes contribute to the observed improvement of proprioceptive precision with age including changes in the regulation of muscle spindle sensitivity, the maturation of the corpus callosum, and the development of central somatosensory pathways. Further work to isolate structures/processes which are involved may prove beneficial for the design and implementation of effective training interventions for children with proprioceptive and associated sensorimotor deficits.

## Author Contributions

JMH-W, I-LY and JK each made substantial contributions to the study design and analysis, as well as aided in drafting, revising and approving the final version of this written work. All authors listed meet the authorship criteria and no one qualified for authorship has been omitted. The first author, JMH-W, collected data, aided in the design of data analysis procedures, performed data analysis generated the first draft of this manuscript and contributed to edits and updates to the document. The second author, I-LY, collected data, aided in the design of data analysis procedures, performed data analysis and contributed to edits and updates of the manuscript. The third author, JK, provided guidance for refining the data collection method, aided in the design of data analysis procedures and contributed to edits and updates of the manuscript.

## Conflict of Interest Statement

The authors declare that the research was conducted in the absence of any commercial or financial relationships that could be construed as a potential conflict of interest.

## Abbreviations

P_diff_: the difference between the match and reference arm angular positions (degrees)
PE: mean of the differences between the match and reference arm angular positions
SDP_diff_: standard deviation of the differences between the match and reference arm angular positions.
